# Supplementary material for: Chinese yuan interest rate swap yields
Source: PLoS One. 2023 Aug 4;18(8):e0289687. doi: 10.1371/journal.pone.0289687 (PMC10403127; doi:10.1371/journal.pone.0289687)
Supplement: S1 Appendix — (DOCX) [file pone.0289687.s002.docx]

**Appendix**

**Table A.1: Granger causality tests**

| Null hypothesis | 2 lags | 3 lags | 4 lags |
| --- | --- | --- | --- |
| ΔCTB3M does not Granger cause ΔCSWAP2Y | 1.85  (0.16) | 0.97  (0.41) | 1.14  (0.34) |
| ΔCSWAP2Y does not Granger cause ΔCTB3M | 6.02***  (0.00) | 3.81**  (0.01) | 3.60**  (0.01) |
| ΔCTB6M does not Granger cause ΔCSWAP2Y | 1.45  (0.24) | 1.16  (0.33) | 1.42  (0.23) |
| ΔCSWAP2Y does not Granger cause ΔCTB6M | 7.74***  (0.00) | 4.80***  (0.00) | 4.77***  (0.00) |
| ΔCTB3M does not Granger cause ΔCSWAP5Y | 1.40  (0.25) | 0.62  (0.60) | 0.94  (0.45) |
| ΔCSWAP5Y does not Granger cause ΔCTB3M | 4.43**  (0.01) | 2.84**  (0.04) | 2.59**  (0.04) |
| ΔCTB6M does not Granger cause ΔCSWAP5Y | 0.95  (0.39) | 0.59  (0.62) | 0.92  (0.46) |
| ΔCSWAP5Y does not Granger cause ΔCTB6M | 5.45**  (0.01) | 3.21**  (0.03) | 3.19**  (0.02) |
| ΔCTB3M does not Granger cause ΔCSWAP10Y | 2.16  (0.12) | 1.26  (0.29) | 1.07  (0.38) |
| ΔCSWAP10Y does not Granger Cause ΔCTB3M | 3.44**  (0.04) | 2.25*  (0.09) | 2.05**  (0.09) |
| ΔCTB6M does not Granger cause ΔCSWAP10Y | 1.33  (0.27) | 0.88  (0.45) | 0.82  (0.52) |
| ΔCSWAP10Y does not Granger cause ΔCTB6M | 3.89**  (0.02) | 2.47*  (0.07) | 2.55**  (0.04) |

Note: F-statistics and probability (in parenthesis) values are reported. ***, **, and * indicates rejection of the null hypothesis at 1 percent, 5 percent, and 10 percent significance levels, respectively.
