# Supplementary material for: Chinese yuan interest rate swap yields
Source: PLoS One. 2023 Aug 4;18(8):e0289687. doi: 10.1371/journal.pone.0289687 (PMC10403127; doi:10.1371/journal.pone.0289687)
Supplement: Attachment — Submitted filename: Review for PLOS One.docx [file pone.0289687.s003.docx]

**Review for PLOS One**

**Reviewed by: Dr. Rabia Arif**

**Chinese Yuan Interest Rate Swap Yields**

The article "Chinese Yuan Interest Rate Swap Yields” focuses on analyzing the dynamics of Chinese Yuan Interest Rate Swap (CNY IRS) yields. The authors examine the volatility and relationships between CNY IRS yields and other macroeconomic variables such as inflation, exchange rate, and money supply. They use daily data from 2014 to 2022 and employ Auto regressive distributed lag (ARDL) technique to estimate the volatility and co-movements between variables more specifically to estimate the impact of short-term swap yields on the long-term interest rate swap yields. Long-term interest swap yields are a valuable tool for monitoring economic trends and informing policy and investment decisions especially for a country like China, given the country's rapidly growing economy and increasing integration into global financial markets. By examining these yields, policymakers and investors can gain valuable insights into the future direction of interest rates and broader economic trends, which can help them to make more informed decisions and manage risk more effectively.

However, I believe that this article should only be accepted after incorporating major changes that are as follows:

1. To make introduction coherent the authors should incorporate following information:

- General picture of China’s economy and link it to the relevance of interest rate swap yields using statistics (include graphs).
- Background of the interest swap yields in China and how have the authors calculated the yields in the literature versus what authors have used in their analysis (if it is any different or same).
- Main results of the study.

2. Expand on literature by including different methodologies that researchers have used to estimate the following relationship and compare it to the one used in papers. Last paragraph should discuss the major contributions of the study. Also cite some studies that have used interest rate swap yields in conducting different analysis for different countries generally and specifically for China.
3. Include theoretical framework.
4. Include empirical equation and explain the methodology in detail.
5. Explain why the specific time period is selected. Also, during this time period a lot of changes have happened in China kindly explain the relevance of those structural changes that may have an impact on the estimated coefficients.
6. Sensitivity analysis is missing from the paper.
7. To check for the reverse causality that may exist in the relation, the authors should report the granger causality test and explain how they have dealt with the problem of reverse causality in the estimations.
8. The tables should be edited properly so that they become reader friendly and it becomes easy to read the dependent variable and independent variables easily. Put down all the other important variables in the notes underneath the tables that were controlled in the regressions.
