# Supplementary material for: Chinese yuan interest rate swap yields
Source: PLoS One. 2023 Aug 4;18(8):e0289687. doi: 10.1371/journal.pone.0289687 (PMC10403127; doi:10.1371/journal.pone.0289687)
Supplement: Attachment — Submitted filename: Response to Reviewers.docx [file pone.0289687.s004.docx]

**PONE-D-23-10931**

**Chinese Yuan Interest Rate Swap Yields**

Dr. Ricky Chee Jiun Chia

Academic Editor

***PLOS ONE***

July 13, 2023

Dear Dr Chia:

We thank the editor and the two referees for their valuable comments. These comments have been extremely useful for us in improving the paper. Hence, we have revised the manuscript, incorporating the referees’ suggestions and addressing the issues they raised. We hope that you will find the revised paper suitable for publication in *PLOS ONE*. The revised parts of the paper are highlighted in yellow.

A separate note responds to each point raised by Referee 1 and Referee 2, stating how we have revised the manuscript in accordance with their comments.

Sincerely,

The authors

*******

**RESPONSE TO REFEREE 1 (ANONYMOUS)**

We thank Referee 1 for the detailed comments. We have revised the paper based on both referees’ valuable suggestions. The revised parts of the paper are highlighted in yellow in the manuscript. Our responses to Referee 1’s comments are provided below.

**Referee 1’s comment 1**: “The authors stated that the null hypotheses (results presented in [Tables] 2a and 2b) for the ADF and the KPSS tests are different, they should briefly explain the differences in the null hypotheses.”

**Authors’ response**: We have explicitly stated the different null hypotheses for the ADF and the KPSS tests in the revised paper (p. 16).

**Referee 1’s comment 2**: “First paragraph of the Econometric Results section: authors should describe the meaning of ‘basis point,’ even in a footnote. This will help those that are not familiar with the financial language.”

**Authors’ response**: We have explained the meaning of ‘basis point’ in the text in the revised paper (p. 19).

**Referee 1’s comment 3:** “The authors should add a brief description of their econometric model.”

**Authors’ response**: We have added a brief description of the econometric model in the revised paper (pp. 10-12).

**Referee 1’s comment 4**: “Including one or two charts and a summary statistic table will help to present their data to the readers.”

**Authors’ response**: We have added three charts. We has also added summary tables (Tables 2A and 2B) that provide summary statistics in the revised paper (pp. 3, 5, 15-16).

**Authors’ additional remarks**: We have extended the dataset in the revised paper. We also addressed Referee 2’s comments.

**RESPONSE TO REFEREE 2 (DR. RABIA ARIF)**

We thank Dr. Rabia Arif (Referee 2) for her detailed comments. We have revised the paper based on both referees’ valuable suggestions. The revised parts of the paper are highlighted in yellow in the manuscript. Our responses to Referee 2’s detailed comments are provided below.

**Referee 2’s general comment on Data availability:** “Have the authors made all data underlying the findings in their manuscript fully available? No.”

**Authors’ response**: We have included the dataset with the paper in an Excel spreadsheet, **S1_Dataset.xlxs**. In the revised version of the paper, we have also included summary statistics and other additional information.

**Referee 2’s comment 1:** “To make introduction coherent the authors should incorporate following information: (1) General picture of China’s economy and link it to the relevance of interest rate swap yields using statistics (include graphs). (2) Background of the interest swap yields in China and how have the authors calculated the yields in the literature versus what authors have used in their analysis (if it is any different or same). (3) Main results of the study.”

**Authors’ response**: We have revised the introduction in the updated version of the paper. The revised introduction provides a brief overview of the Chinese economy and recent developments therein. We also added some background information on the use of CNY swaps and summarized the main results of the study in the introduction, as suggested by the referee (pp. 2-5).

**Referee 2’s comment 2**: “Expand on literature by including different methodologies that researchers have used to estimate the following relationship and compare it to the one used in papers. Last paragraph should discuss the major contributions of the study. Also cite some studies that have used interest rate swap yields in conducting different analysis for different countries generally and specifically for China.”

**Authors’ response**: We discuss the different approaches that researchers have used in their attempts to model the dynamics of swap yields. We argue that the Keynesian perspective is a fruitful approach to modeling the dynamics of swap yields. We cite several studies that have modeled interest rate swap yields in different countries. An extensive research in the major databases, including JSTORE, ECONLIT, Google Scholar, PROQUEST, SSRN, and so forth, shows that there are no econometric studies of CNY-denominated interest rate swap yields. This paper fills a critical gap in the literature (pp. 6-10)

**Referee 2’s comment 3**: “Include theoretical framework.”

**Authors’ response**: We have provided a theoretical framework and illustrated the theoretical framework with a simple quantitative model of the dynamics of swap yields based on key macroeconomic variables. All the additions are presented in yellow highlight (pp. 11-12).

**Referee 2’s comment 4**: “Include empirical equation and explain the methodology in detail.”

**Authors’ response**: In the revised paper we have included the empirical equations and explained the methodology in detail (pp. 18, 24).

**Referee 2’s comment 5**: “Explain why the specific time period is selected. Also, during this time period a lot of changes have happened in China. Kindly explain the relevance of those structural changes that may have an impact on the estimated coefficients.”

**Authors’ response**: The time period is based on: (1) the introduction and widespread use of CNY-denominated interest rate swaps for both hedging and speculating on interest rate risks by domestic and overseas parties, and (2) the availability of the data. **Please note that the dataset has been extended in the revised version to include the latest data available as of June 2023.**

**Referee 2’s comment 6**: “Sensitivity analysis is missing from the paper.”

**Authors’ response**: We have provided robust checks to our estimates by using a different set of independent variables. Specifically we have used 6-month Treasury bills’ rates instead of 3-month Treasury bills’ rates for the short-term interest rate; total inflation instead of core inflation; Shenzhen equity price index instead of Shanghai equity price index; and the exchange rate of the Chinese yuan against the euro instead of the US dollar. The econometric results are quite similar across the swap yield curve. Moreover, it should be pointed out that for each of the dependent variables, namely the 2-year, 5-year, and 10-year swap yields, three different models are estimated. All of these models show that the short-term interest rate has a statistically significant and economically important effect on the swap yield. This implies that the econometric findings are robust to changes in the functional forms of the regression models. The results of the sensitivity analysis are presented in Tables 6A, 6B, and 6C (pp. 24-27).

**Referee 2’s comment 7**: “To check for the reverse causality that may exist in the relation, the authors should report the granger causality test and explain how they have dealt with the problem of reverse causality in the estimations.”

**Authors’ response**: We have undertaken Granger causality tests. The test results are presented in Appendix A (p. 29). We also discuss how the ARDL models address reverse causality in our estimations (p. 28).

**Referee 2’s comment 8**: “The tables should be edited properly so that they become reader friendly and it becomes easy to read the dependent variable and independent variables easily.”

**Authors’ response:** We have revised the tables by breaking them into three separate tables (see Tables 5A, 5B, and 5C) for different swap maturity tenors to present the econometric results (pp. 20-22).

**Authors’ additional remarks**: We have extended the dataset. We also addressed Referee 1’s comments.
